# Organic/Inorganic Species Synergistically Supported Unprecedented Vanadomolybdates

**DOI:** 10.3390/molecules27217447

**Published:** 2022-11-02

**Authors:** Tian Chang, Di Qu, Bao Li, Lixin Wu

**Affiliations:** State Key Laboratory of Supramolecular Structure and Materials, College of Chemistry, Jilin University, Changchun 130012, China

**Keywords:** polyoxometalate, vanadomolybdate, triol ligand, covalent modification

## Abstract

Vanadomolybdates (VMos), comprised of Mo and V in high valences with O bridges, are one of the most important types of polyoxometalates (POMs), which have high activity due to their strong capabilities of gaining/losing electrons. Compared with other POMs, the preparation of VMos is difficult due to their relatively low structural stability, especially those with unclassical architectures. To overcome this shortcoming, in this study, triol ligands were applied to synthesize VMos through a beaker reaction in the presence of V_2_O_5_, Na_2_MoO_4_, and organic species in the aqueous solution. The single-crystal X-ray diffraction results indicate that two VMo clusters, Na_4_{V_5_Mo_2_O_19_[CH_3_C(CH_2_O)_3_]}∙13H_2_O and Na_4_{V_5_Mo_2_O_19_[CH_3_CH_2_C(CH_2_O)_3_]}∙13H_2_O, with a similar architecture, were synthesized, which were both stabilized by triol ligand and {MoO_6_} polyhedron. Both clusters are composed of five V ions and one Mo ion in a classical Lindqvist arrangement with an additional Mo ion, showing an unprecedented hepta-nuclear VMo structure. The counter Na^+^ cations assemble into one-dimensional channels, which facilitates the transport of protons and was further confirmed by proton conductivity experiments. The present results provide a new strategy to prepare and stabilize VMos, which is applicable for developing other compounds, especially those with untraditional architectures.

## 1. Introduction

**Figure 1:**
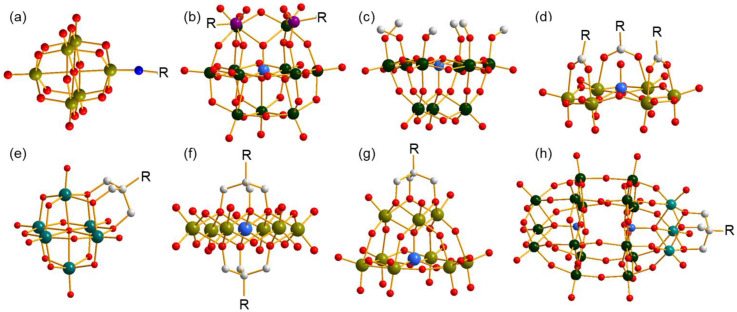
Ball-and-stick representation of several classical organo-functionalized clusters. (**a**) Imido-functionalized Lindqvist-type polyanion, (**b**) organophosphorus-functionalized lacunary Keggin-type polyanion, (**c**) methanol-functionalized lacunary Keggin-type polyanion, (**d**) carboxyl-functionalized Anderson-like-type polyanion; triol-ligand-functionalized (**e**) Lindqvist-type, (**f**) Anderson-type, (**g**) lacunary Keggin-type, and (**h**) Dawson-type polyanions. All H atoms are omitted for clarity. R represents the functional group. Dark cyan ball: V, olive ball: Mo, dark green ball: W, purple ball: P, light grey ball: C, red ball: O, blue ball: N, light blue ball: heteroatom.

Organic species covalent functionalization on polyoxometalates (POMs) is an efficient method of constructing POMs that possess novel architectures and properties, and results in abundant achievements in the fields of organic and inorganic hybrids as well as materials science [1,2]. To date, several strategies have been developed in accordance with the structural features of POMs, and the classical structures are shown in Figure 1. By utilizing the strong Mo≡N bond, some N-containing organic molecules are capable of being anchored on a Lindqvist-type cluster [Mo_6_O_19_]^2−^ (Figure 1a) [3,4]. In some lacunary POMs, P, Si, Sn, or Ge occupy the vacant position and introduce organically functional groups through P–C, Si–C, Sn–C, or Ge–C bonds (Figure 1b) [5]. As a comparison, modification through M–O–C (M = Mo, W, V) is a more convenient and more common method which provides volatile connection modes between organic and inorganic parts and is applicable to different types of POMs. The simplest modification through an M–O–C bond is that methanol or ethanol molecules replace bi- or tri-bridging O atoms of clusters, resulting in partially substituted POMs, which is applicable to nearly all types of POMs (Figure 1c) [6]. Beyond a single M–O–C bond, carboxyl is also a good candidate to covalently bond on the cluster through two M–O–C bonds sourced from one molecule, which provides a stronger combination (Figure 1d) [7,8]. In addition, triol ligands have the ability to supply three M–O–C bonds linking to the same C center through three methene units, which largely improves the stability of the formed complexes, and therefore has obtained rapid progress in the last two decades [9,10,11]. To date, Lindqvist-(Figure 1e), Anderson-(Figure 1f), Keggin-(Figure 1g), and Dawson-type clusters (Figure 1h) have all been involved in the successful modification by triol ligands with various architectures, which express excellent properties and various applications [12,13,14]. The unique coordination behavior of the triol ligand endows its ability to stabilize structures and makes it a good candidate for building POMs with novel or metastable clusters.

Vanadomolybdates (VMos), as one of the important branches of POM chemistry, have attracted extensive attention due to their diverse structures and multi-functions in the fields of electrochemistry, magnetism, and catalysis [15,16,17]. Compared with other POMs, VMos have a stronger ability to gain or lose electrons, and therefore possess a higher activity, resulting in their excellent properties [18,19]. On the other hand, the structures of VMos have relatively low stability, especially those of lacunary species, which need additional components to support their architectures. For instance, with the aid of a transition metal ion dimer {Mn_2_}, two unstable trivacant Keggin-type VMo clusters were linked to form a sandwich structure, which shows an unusual ferromagnetic coupling [20]. Another strategy for preparing VMos is utilizing the organic species, which are covalently anchored on the clusters and stabilize the obtained hybrids. A classical case was reported by Wei et al., where an unprecedented {V_4_Mo_3_} was synthesized in the presence of anilines as stabilizers, which replaced six terminal or bi-bridging O atoms of the {Mo_3_} cluster [21]. With a similar strategy, an Anderson-type VMo cluster was also isolated, in which two triol ligands replaced all hydroxyls surrounding the heteroatom V, resulting in doubly decorated hybrids [22]. In another example, triethanolamine was applied to stabilize a {VMo_6_O_25_} cluster, forming an organic component single-functionalized product [23]. We have also made a contribution in this field showing that a triol ligand is used to anchor on the top position of [VMo_9_O_34_]^9−^ cluster with the maintaining of the vacant sites, which are considered to be more active in the reaction [24]. These results indicate that organically covalent modification is an efficient method to prepare VMos, especially those without stable structures in the isolated state. More importantly, with the help of the coordination ability of organic species, VMos with novel structures are expectable.

During our investigation on triol-ligand-modified VMos, we discovered one type of novel Lindqvist-type derivative with V_2_O_5_, Na_2_MoO_4_, and triol ligand as reactants. In this work, the synthesis and structural characterization of Na_4_{V_5_Mo_2_O_19_[CH_3_C(CH_2_O)_3_]}∙13H_2_O (**1**) and Na_4_{V_5_Mo_2_O_19_[CH_3_CH_2_C(CH_2_O)_3_]}∙13H_2_O (**2**) are presented. The polyanions of compounds **1** and **2** are isostructural except the terminal group of the triol ligand, which can both be seen as a {MoO_6_} polyhedron attached to a Lindqvist cluster by sharing three O atoms. As far as we know, this type of hepta-nuclear VMo has never been reported in the POM family. The results presented here would provide a new route for preparing triol-ligand-modified VMos with unusual architectures.

## 2. Results and Discussion

### 2.1. Synthesis of Compounds ***1*** and ***2***

In the presence of the triol ligand, V_2_O_5_ and Na_2_MoO_4_∙2H_2_O were added to water, which resulted in the formation of a light brown solution under heating conditions. Crystals of compounds **1** and **2** were obtained by standing the solutions for a week. Several factors are supposed to play important roles in the successful synthesis of compounds. Firstly, the molar ratio of V/Mo in the reactants is one such factor. Only when the molar ratio of V/Mo is in the range of 5:2 and 5:3 can the targeted products be obtained. If a higher percentage of V exists in the reactants, a triol-ligand-modified hexavanadate cluster is obtained, which has the same architecture as that reported in the literature [25]. If reactants with lower percentages of V are used, some VMo clusters are obtained and most of them cannot provide high-quality crystals for further accurate determination of their structures. Secondly, the control of pH is another essential factor. When the pH of the reaction solution is controlled between 4–5, the quality of the crystals is good for giving high-quality X-ray diffraction data for analysis. If the pH decreases to 3–4, the quality of the crystals becomes poor and is not very stable in air, which quickly degenerates into powder after the crystals leave the mother liquid due to weathering. In a more acidic environment with a smaller pH value, crystals cannot be obtained. On the other hand, when pH is higher than 5, the solubility of the reactants in the water becomes poor, and nearly no reaction occurs. In addition, the amount of triol ligand seems to have so little influence on the product that excessive organic species cannot change its number on the cluster, and only one triol ligand remains attached to the polyanion. On the other hand, the presence of the triol ligand is the essential factor for the formation of the present architecture. We have also conducted experiments under similar conditions without a triol ligand, and as a result, only some VMo species with usual structures were obtained. All these factors indicate that the compounds presented here are only successfully synthesized under relatively harsh conditions, which need good modulation to the reactions.

### 2.2. Structures of Compounds ***1*** and ***2***

**Figure 2:**
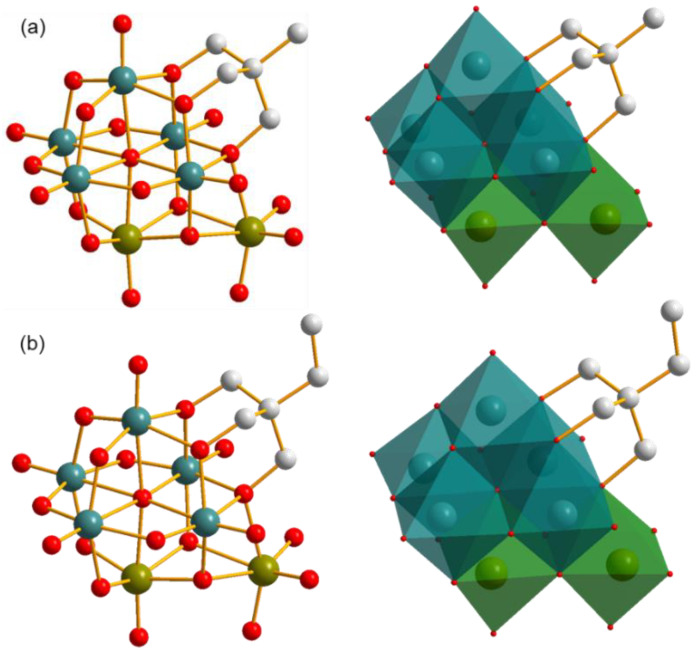
Polyanionic structures of compounds (**a**) **1** and (**b**) **2** in ball-and-stick representation (left) and combined ball-and-stick and polyhedron representation (right). All H atoms are omitted for clarity. Dark cyan ball: V, olive ball: Mo, light grey ball: C, red ball: O, dark cyan polyhedron: {VO_6_}, olive polyhedron: {MoO_6_}.

Single crystal X-ray diffraction analysis reveals that compounds **1** and **2** possess very similar architectures except the difference of the terminal groups of triol ligand, methyl for compound **1** and ethyl for compound **2**, as shown in Figure 2. Here, compound **1** is used as a representative for illustrating their structural characteristics.

#### 2.2.1. Inorganic Architecture

The inorganic architecture of the polyanion of compound **1** is composed of five {VO_6_} and two {MoO_6_} polyhedra in the edge-sharing style. A Lindqvist-type cluster including five {VO_6_} and one {MoO_6_} polyhedra can be distinguished with a μ_6_-O as the center, as shown in Figure S1a. The other {MoO_6_} polyhedron attaches to this cluster through three μ_2_-O atoms in a face-sharing style, in which one O atom is from the V–O–V unit and the other two O atoms are sourced from V–O–Mo units. This connection makes the two {MoO_6_} polyhedra neighbors, and therefore the polyanion of compound **1** can also be seen as the combination of a mono-lacunary Lindqvist {V_5_} cluster with a metal dimer {Mo_2_} in the presence of five shared O atoms, as shown in Figure S1b. In addition, all bond lengths and angles in the cluster are in the normal ranges, which are listed as Tables S1–S4 in the Supporting Information.

The unique combination of compound **1** results in an unreported atomic V/Mo ratio of 5:2. As an important sub-class of POMs, many VMos have been synthesized and reported, which possess various atomic V/Mo ratios. The change of atomic V/Mo ratio has an obvious influence on the architectures of the clusters, as well as their properties, which generally originates from the following aspects: (1) The V ion is used as a heteroatom in VMos, such as 1:6 for an Anderson-type POM, 1:12 for a Keggin-type POM, and 2:18 for a Dawson-type POM. It can also be applied in the lacunary cluster, such as 1:9 for a trivacant Keggin-type POM. (2) The V ion is used to replace one or more addenda atoms of clusters. For isopolyoxometalates, the Lindqvist-type [Mo_6_O_19_]^2−^ can be substituted by one or two V ions to form a cluster with 1:5 or 2:4 atomic V/Mo ratios, while for octamolybdates, the substitution of one or two V ions results in products having 1:7 or 2:6 atomic V/Mo ratios, which derivatives have been widely investigated. For the heteropolyoxometalates, there are more possibilities due to the increase of the number of addenda atoms, which generate a series of VMos derivatives. (3) A V ion is used to build VMo clusters with nonclassical architectures. With this strategy, {VO_4_}/{VO_6_} can combine with {MoO_6_} in various manners, and with the aid of other organic or inorganic species, more clusters with novel structures are obtained. Through the above-mentioned strategies, VMos with different atomic V/Mo ratios have been achieved, which are briefly summarized in Table 1. It should be noted that partial V^V^ ions can be reduced to V^IV^ ions, which enriches the diversity of the structures. In the present case, by utilizing the strong coordination ability of the triol ligand, an extra {MoO_6_} octahedron is introduced into the [V_5_MoO_19_]^7−^ polyanion, resulting in the formation of architecture with an unreported atomic V/Mo ratio of 5:2. This result also proves that the atomic V/Mo ratio has an important influence on the structure of the cluster, which can be tuned and applied for finding new VMo species.

#### 2.2.2. The Decoration of Triol Ligand on the Cluster

As mentioned above, compounds **1** and **2** possess a unique inorganic architecture, as well as an unreported atomic V/Mo ratio. Another feature of these two compounds is that a triol ligand attaches to the inorganic cluster, which plays a key role in the formation of polyanions. As is well known, bare Lindqvist-type polyoxovanadates (POVs) are not stable, and they can only be achieved in the presence of alcohols replacing one or more μ_2_-O atoms to reduce the charge density of the surface. The used alcohols can be methanol and ethanol, as well as a triol ligand, which can provide a strong coordination environment and anchors on the cluster up to four times. As the derivatives of Lindqvist-type POVs, triol ligand is also the essential factor for the successful preparation of compounds **1** and **2**. It is the same with those in the Lindqvist-type POVs; the triol ligands in compounds **1** and **2** also replace three μ_2_-O atoms of V–O–V units and cover on a {V_3_} cluster. The decoration of triol ligand has an obvious influence on the V–(μ_2_-O) bond lengths concerned, which possess an average value of 2.038 Å (in compound **1**). As a comparison, other V–(μ_2_-O) bonds, which are not involved in the coordination with triol ligands, have a relatively shorter average bond length of 1.862 Å. A similar phenomenon is also found in triol-ligand-decorated trivacant Kegging-type cluster, showing the strong coordination ability of triol ligand [24].

A more interesting feature of the polyanions of compounds **1** and **2** is that the present cluster can be seen as the triol ligand and the {MoO_6_} polyhedron co-supported [V_5_MoO_19_]^7−^ polyanion. As shown in Figure S2a, both the triol ligand and {MoO_6_} polyhedron share three O atoms with the [V_5_MoO_19_]^7−^ cluster. This structure is different from the two-triol-ligand covalently decorated POVs [47]. As shown in Figure S2b,c, the triol ligands can anchor on the Lindqvist-type POVs in cis or trans configurations by replacing six different μ_2_-O atoms, while in the present case, the triol ligand shares an O atom with the additional {MoO_6_} polyhedron, resulting in their combination with the [V_5_MoO_19_]^7−^ cluster through a total of five shared O atoms. In addition, it should be noted that all three terminal O atoms of {MoO_6_} polyhedron are involved in the formation of Mo=O double bonds, with the bond lengths of 1.730, 1.730, and 1.744 Å, which are different from those in the triol-ligand-decorated heptavanadate cluster, with one water molecule serving as a terminal group with a much longer V–O bond length of 1.995 Å [48]. The triol ligand modification on the cluster also influences the packing model of the polyanions. As shown in Figure S3, a double layer comprised of polyanions forms, in which triol ligands face opposite directions towards the inside of the layer. This packing model can reduce the exposure possibility of methyl groups in an aqueous solution, which benefits the minimalization of interface energy and the crystallization of the compounds.

#### 2.2.3. The Assembly Structure of Cations in Compounds **1** and **2**

Both compounds **1** and **2** crystallize with Na^+^ as counter cations, and four crystally independent Na^+^ ions exist in every asymmetric unit. Taking compound **1** as the example, Na1, Na2, and Na3 are located on the symmetric plane and therefore possess the site occupancy of 0.5. For Na4, though it is not located at any symmetry element, and has an apparent site occupancy of 1, it is treated as a disorder due to the following properties: (1) Na4 and its symmetric atom Na4′ have a distance of 2.128 Å, which is much shorter than that between two normal sodium cations with a distance over 3.0 Å, and is even shorter than the Na–O bond length with a general value over 2.3 Å. The abnormally short distance between Na4 and Na4′ indicates that the two Na^+^ ions should exist alternately, showing a disorder in space. (2) Na4 has a thermal displacement parameter over 0.1, which is much higher than those of Na_1_, Na_2_, and Na_3_ with an average value of about 0.04. After the disorder treatment, the thermal displacement parameter of Na4 decreases to about 0.04 and remains stable in the anisotropic state after several refinement cycles. (3) The whole polyanion has a total charge of −4, which needs two positive charges in the asymmetric unit for charge neutralization. Additionally, in the presence of three Na^+^ ions with half site occupancies, the fourth one should also occupy the 0.5 site, which is also in accordance with the above analysis conditions. Finally, the elemental analysis result of Na also supports this treatment. 

**Figure 3:**
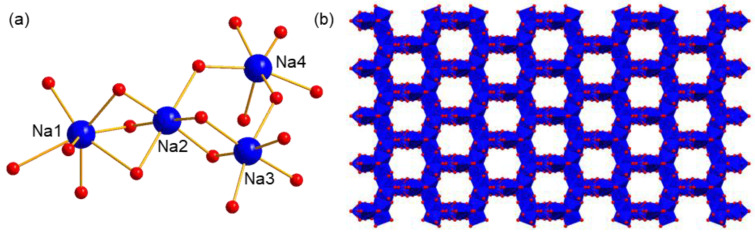
(**a**) The coordination environments of four independent Na^+^, and (**b**) the packing model of counter cation Na^+^ in compound **1**. The polyanions are omitted for clarity. Blue ball: Na, red ball O, blue polyhedron: {NaO_6_} or {NaO_7_}.

Four Na^+^ ions express different coordination environments as well as coordination numbers (Figure 3a). Na1 is coordinated by six water molecules and one terminal O atom of polyanion, showing a seven-coordination type. Na2, Na3, and Na4 are all in a six-coordination type, in which all the coordination sites of Na2 and Na3 are occupied by water molecules. As a comparison, the coordination environment of Na4 is completed by five water molecules and one O atom from polyanion. One important feature of these Na^+^ ions is that the coordinated water molecules are generally shared by two or three cations, resulting in their possessing wide edge- or face-connection with each other. This feature also facilitates the formation of the extended structure. As shown in Figure 3b, a regular honeycomb structure formed by Na^+^ ions and its coordinating O atoms can be obtained, showing one-dimensional (1D) channels along the c axis. Here, to better exhibit the architecture of the packing of Na^+^ ions, polyanions are omitted for clarity except for two O atom links, with Na^+^ being kept. The passageways constructed here are helpful for the fast migration of ions, which can be used as a proton conductor. 

#### 2.2.4. FT-IR, XPS and TGA Curves of Compounds **1** and **2**

Except for single crystal X-ray diffraction analysis, the structures of compounds **1** and **2** are also confirmed by FT-IR spectra. As shown in Figure S4a, the FT-IR spectrum of compound **1** in the low wavenumber region expresses characteristic vibration of the inorganic cluster sourced from V–O, Mo–O, and Mo=O. The existence of wavenumbers over 1000 cm^−1^ can be ascribed to the vibrations of C–O, C–H, and H–O, showing the occurrence of organic components in the compound. Compound **2** has a similar FI-IR pattern as that of compound **1**, which is shown in Figure S4b.

XPS was used to identify the bond valence of Mo and V in the clusters. For compound **1**, as shown in Figure S5a, the binding energy peaks located at 235 and 232 eV are sourced from Mo^VI^3d_3/2_ and Mo^VI^3d_5/2_, indicating that the bond valence of Mo is +6. The bond valence of V is determined in Figure S5b, in which two binding energy peaks at 517 and 524 eV are V^V^2p_1/2_ and V^V^2p_3/2_, respectively, showing a +5 bond valence for V. As shown in Figure S6, the XPS spectra of compound **2** are similar to those of compound **1**, which also indicate the existence of Mo^VI^ and V^V^ in the cluster. 

To verify the purity of the prepared compounds, the powder X-ray diffraction (PXRD) patterns of compounds **1** and **2** was checked. As shown in Figure S7, the as-synthesized and simulated PXRD patterns are similar for each compound, indicating that the powder products maintained the same architectures as those in the single crystal state.

The thermal stability of compounds **1** and **2** were evaluated through TGA analysis. As shown in Figure S8, both compounds **1** and **2** quickly lose their lattice water molecules in the range of room temperature to 100 °C. Additionally, the organic species leave the cluster, followed by the decomposition of polyanion, showing the supporting role of triol ligand in the cluster.

#### 2.2.5. Stability of Compounds **1** and **2** in Aqueous Solution

The existing states of compounds **1** and **2** in an aqueous solution were examined using ^1^H NMR spectra. As shown in Figure S9, compound **1** is not very stable in the aqueous solution and decomposition occurred in the process of dissolving. The signals belonging to the free triol ligand appeared at the beginning and increased over time. Calculated from the integration value of the peaks, the initial decomposition ratio is about 6.6%, and reaches 7.9% after 3 days. The different chemical environments of methene groups in compound **1** generate the splitting of the signal, showing double peaks at around 4.9 ppm. Compound **2** shows a similar behavior to that of compound **1** in an aqueous solution (Figure S10). The difference is that compound **2** seems to have a relatively higher stability, and only 2.1% decomposes at the beginning, reaching 6.2% after 3 days. It should be noted that the decomposition process can be accelerated by the addition of acid or base.

### 2.3. Proton Conductivity of Compounds ***1*** and ***2***

**Figure 4:**
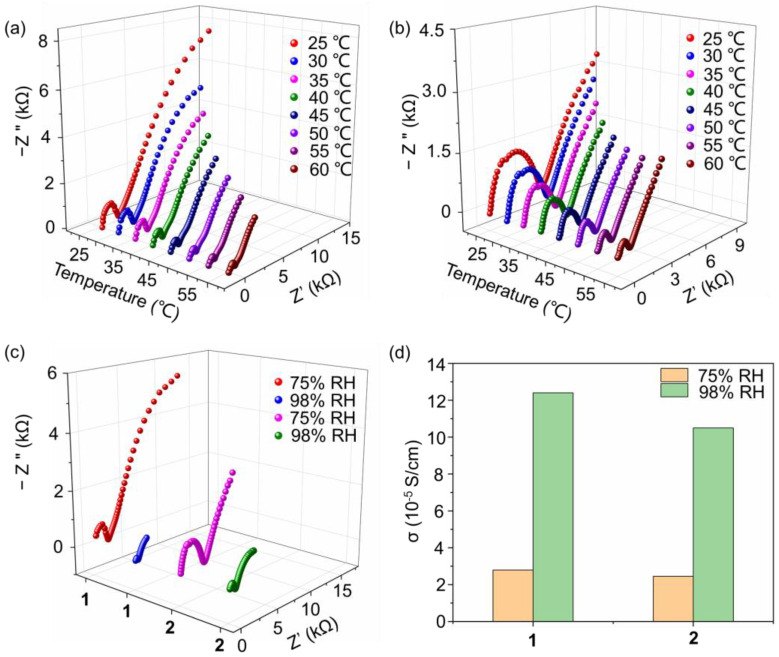
Nyquist plots of compounds (**a**) **1**, and (**b**) **2** at various temperatures and 75% RH; (**c**) Nyquist plots of compounds **1** and **2** under the conditions of 75% and 98% RH at 30 °C; (**d**) proton conductivity of compounds **1** and **2** under different conditions.

Proton-conducting materials play essential roles in fuel cells, which have seen rapid progress due to the demand for clean energy. For building high-proton-conductivity materials, an extensive hydrogen bonding network is needed, which accelerates the transport of the proton and induces energy loss. POMs are good candidates for proton-conducting materials due to their abundant O atoms on the surface of the cluster, which can serve as hydrogen bonding acceptors. Here, the proton conductivity of compounds **1** and **2** under different temperatures are investigated in the condition of a controlled 75% relative humidity (RH). As shown in Figure 4a, with the temperature changing from 25 to 60 °C, the proton conductivity of compound **1** increased from 2.25 × 10^−5^ to 7.74 × 10^−5^ S cm^−1^. Based on the proton conductivities at various temperatures, the activation energy (E_a_) of the proton conductivity of compound **1** was evaluated. As shown in Figure S11a, a linear fitness can be obtained with the ln(σT) as longitudinal ordinate and 1/T as horizontal ordinate, from which the activity energy E_a_ is calculated as 0.32 eV. The relatively low E_a_ value (smaller than 0.4 eV) indicates that the proton conduction process in compound **1** is ascribed to a Grotthuss mechanism. This result is also in accordance with the crystal structure described above, in which a 1D channel exists and facilitates the transfer of protons. In addition, the water molecules on the wall of the channel also promote proton transport. The proton conductivity behavior of compound **2** was also investigated to evaluate the influence of the terminal group of the triol ligand. As shown in Figure 4b, in the range of 25 to 60 °C, the proton conductivity increases from 1.97 × 10^−5^ to 7.48 × 10^−5^ S cm^−1^. It can be seen that compounds **1** and **2** have similar proton conductivity, which sources their similar crystal structures as well as packing models. The slight decrease of proton conductivity of compound **2** compared to that of compound **1** is ascribed to originate from the slightly higher hydrophobicity of ethyl to methyl. In addition, the E_a_ of proton conduction for compound **2** was calculated as 0.36 eV (Figure S11b), which also indicates a Grotthuss mechanism.

We further investigated the influence of RH on proton conductivity. As shown in Figure 4c, when the RH increases to 98%, the proton conductivity of compound **1** increases to 1.24 × 10^−4^ S cm^−1^ at 30 °C, showing a 4.4 times larger elevation compared with that under 75% RH. In a higher RH condition, the number of dissociated water molecules increases and promotes the transport of protons in the 1D channel through the stable hydrogen bonding network. Compound **2** also shows similar proton conductivity behaviors and has a value of 1.05 × 10^−4^ S cm^−1^ at 30 °C and 98% RH, which also shows a 4.3 times improvement. By collecting proton conductivity under different conditions, as shown in Figure 4d, it can be concluded that RH has a more obvious influence than that of temperature. In addition, as listed in Table S5, we have also collected the recent results of the proton conductivity of POMs, showing that compounds **1** and **2** possess a relatively high performance.

## 3. Materials and Methods

### 3.1. Instruments and Materials

All chemicals used here were purchased from Aladdin and used directly. All reactions were conducted with double-distilled water as solvent. C and H elemental analysis was made on a vario MICRO cube from the Elementar Company of Germany. Elemental analysis of V, Mo, and Na was conducted on a PLASMA-SPEC (I) inductively coupled plasma atomic emission spectrometer. FT-IR spectra were obtained from a Bruker Vertex 80v spectrometer in KBr pellets, in which the detector was DTGS and the resolution was 4 cm^−1^. Thermal stability of compounds was evaluated by a Q500 Thermal Analyzer (New Castle TA Instruments, New Castle, DE, USA) with a flowing N_2_ atmosphere and a heating rate of 10 °C∙min^−1^. XPS data were acquired on an ESCALAB-250 spectrometer with a monochromic X-ray source (Al Kα line, 1486.6 eV). The charging shift of XPS was corrected at a binding energy of C1s at 284.6 eV. Electrochemical impedance spectroscopy was recorded on a Solartron 1260 A impedance analyzer. Powder X-ray diffraction data were recorded on a Rigaku SmartLab X-ray diffractometer with a Cu K_α_ radiation at a wavelength of 1.54 Å. ^1^H NMR spectra were obtained from a Bruker AVANCE 500 MHz spectrometer. The proton conductivities of samples were calculated according to the following equation:

σ = l/SR

where σ is the proton conductivity (S/cm), and l, and S, and R are the thickness, area, and resistance of the sample, respectively.

Single-crystal X-ray diffraction data of compounds **1** and **2** were collected on a Bruker D8 VENTURE diffractometer with graphite-monochromated Mo Kα (λ = 0.71073 Å) at 293 K. Both crystals were solved by *SHELXT* and refined by full-matrix-least-squares fitting for *F*^2^ using the Olex^2^ software. All non-H atoms were refined with anisotropic thermal parameters. A summary of the crystallographic data and structural refinements for compounds **1** and **2** is listed in Table 2. The files with CCDC numbers 2206198 and 2206199 contain the supplementary crystallographic data for this paper. These data are available free of charge from www.ccdc.cam.ac.uk/data_request/cif (accessed on 9 September 2022), or by emailing data_request@ccdc.cam.ac.uk, or by contacting The Cambridge Crystallographic Data Centre, 12 Union Road, Cambridge, CB2 1EZ, UK.

### 3.2. Synthesis of Compounds ***1*** and ***2***

#### 3.2.1. Synthesis of Na_4_{V_5_Mo_2_O_19_[CH_3_C(CH_2_O)_3_]}∙13H_2_O (1)

Na_2_MoO_4_∙2H_2_O (0.88 g, 2.00 mmol) and V_2_O_5_ (0.45 g, 2.50 mmol) were mixed in 10 mL of deionized water, and then CH_3_C(CH_2_OH)_3_ (0.24 g, 2.00 mmol) was added under stirring. Concentrated HCl was used to adjust the pH of the mixture to 4~5, which was further heated to 60 °C and maintained for 4 h. After a hot filtration process, a light brown solution was obtained, which generated light brown crystals after about one week at room temperature with a yield of 54.1% based on Mo. Elemental analysis calcd. (%) for Na_4_{V_5_Mo_2_O_19_[CH_3_C(CH_2_O)_3_]}∙13H_2_O (Mw = 1193.87 g mol^−1^): C 5.03%, H 2.96%, Na 7.70%, V 21.33%, and Mo 16.07%; found C 5.06%, H 2.98%, Na 7.71%, V 21.50%, and Mo 16.01%.

#### 3.2.2. Synthesis of Na_4_{V_5_Mo_2_O_19_[CH_3_CH_2_C(CH_2_O)_3_]}∙13H_2_O (2)

The synthetic procedure of compound **2** was similar to that of compound **1**, except that the triol ligand was replaced by CH_3_CH_2_C(CH_2_OH)_3_ (0.27 g, 2.00 mmol) and the yield was 55.2% based on Mo. Elemental analysis calcd. (%) for [Na_4_{V_5_Mo_2_O_19_[CH_3_CH_2_C(CH_2_O)_3_]}∙13H_2_O (Mw = 1207.89 g mol^−1^): C 5.97%, H 3.09%, Na 7.61%, V 21.09%, and Mo 15.89%; found C 5.89%, H 3.05%, Na 7.55%, V 21.03%, and Mo 15.94%.

## 4. Conclusions

In conclusion, triol-ligand and {MoO_6_} polyhedron co-decorated [V_5_MoO_19_]^7−^ polyanion is constructed with different terminal groups. The prepared compounds present a new type of derivative of Lindqvist-type clusters, which have never been reported previously. The organic and inorganic parts both attach to the main polyanion by replacing three O atoms in a *cis* conformation, in which one O atom is shared by three components and further improves the stability of the cluster. This manner of combination is different from those two triol-ligand-decorated Lindqvist-type clusters, in which no O atoms are shared by organic species. Another feature of the prepared compounds is that they express a new type of VMos with an unreported atomic ratio of 5:2. In addition, the Na^+^ cations in compounds assemble into 1D channels, whose walls are full of water molecules. The further proton conductivity experiments show that this structure facilitates the transport of protons, which bears a Grotthuss mechanism for both compounds. 

## Figures and Tables

**Table 1:** Summary of atomic V/Mo ratios in some classical VMos.

Atomic V/Mo Ratio *^a^*	Formula of Polyanion *^b^*	Valence of V	Valence of Mo	Ref.
1:4	[VMo_4_O_17_]^5−^	+5	+6	[26]
1:5	[VMo_5_O_19_]^3−^	+5	+6	[27]
1:6	[VMo_6_O_24_]^6−^	+5	+6	[16]
1:7	[VMo_7_O_26_]^5−^	+5	+6	[15]
1:9	{VMo_9_O_31_[CH_3_C(CH_2_O)_3_]}^6−^	+5	+6	[24]
1:11	[P(VMo_11_)O_40_]^4−^	+5	+6	[28]
1:12	[VMo_12_O_40_]^3−^	+5	+6	[29]
2:2	[V_2_O_2_(μ-MeO)_2_(μ-MoO_4_)_2_(4,4′-tBubpy)_2_]	+5	+6	[30]
2:4	[V_2_Mo_4_O_19_]^4−^	+5	+6	[31]
2:6	[V_2_Mo_6_O_26_]^6−^	+5	+6	[32]
2:8	[HV_2_Mo_8_O_32_]^5−^	+5	+6	[26]
2:10	[HV_2_Mo_10_O_38_]^5−^	+5	+6	[26]
2:16	[V_2_Mo_16_O_58_]^10−^	+5	+6	[33]
2:18	[V_2_Mo_18_O_62_]^6−^	+5	+6	[34]
2:22	[Fe_5_CoMo_22_V_2_O_87_(H_2_O)]}^12−^	+5	+6	[35]
3:3	[V_3_Mo_3_O_16_(C_5_H_9_O_3_)]^2−^	+5	+6	[36]
3:9	[V_3_Mo_9_O_38_]^7−^	+5	+6	[26]
3:10	[V(V^IV^V^V^Mo_10_O_40_)]^6−^	+4, +5	+6	[37]
3:17	[H_2_V^IV^Mo_17_O_54_(V^V^O_4_)_2_]^6−^	+4, +5	+6	[38]
4:3	[V_4_Mo_3_O_14_(NAr)_3_(μ_2_-NAr)_3_]^9−^	+5	+6	[21]
4:8	[As_2_V_4_Mo_8_AsO_40_]^5−^	+5	+6	[39]
5:2	{V_5_Mo_2_O_19_[CH_3_C(CH_2_O)_3_]}^4−^	+5	+6	This work
5:4	[V_5_Mo_4_O_27_]^5−^	+5	+6	[40]
5:8	[V_5_Mo_8_O_40_]^7−^	+5	+6	[41]
6:57	[V_6_Mo_57_O_183_(NO)_6_(H_2_O)_18_]^6−^	+5	+6	[42]
7:8	[(V^V^Mo_8_V_4_^IV^O_40_)(V^IV^O)_2_]^7−^	+4, +5	+6	[43]
7:11	[V^V^_5_V^IV^_2_Mo_11_O_52_(SeO_3_)]^7−^	+4, +5	+6	[44]
8:2	[V_8_Mo_2_O_28_]^4−^	+5	+6	[26]
8:4	[V_8_Mo_4_O_36_]^8−^	+5	+6	[45]
9:1	[V_9_MoO_28_]^5−^	+5	+6	[31]
9:8	[(V^V^Mo^VI^_4_Mo^V^_4_V_4_^IV^O_40_)(V^IV^O)_4_]^7−^	+4, +5	+6	[43]
10:12	[Mo_12_V_10_O_58_(SeO_3_)_8_]^10−^	+5	+6	[44]
14:16	[V_12_^IV^V^V^_2_Mo_16_O_84_]^14−^	+4, +5	+6	[46]

*^a^* The atomic V/Mo ratios used here are the same with as those in formulas without an approximate process. *^b^* The apparent sequence of V and Mo in the formulas of polyanions are unified.

**Table 2:** The summary of crystal data and structural refinements for compounds **1** and **2**.

Item	Compound 1	Compound 2
Formula	Na_4_{V_5_Mo_2_O_19_[CH_3_C-(CH_2_O)_3_]}∙13H_2_O	Na_4_{V_5_Mo_2_O_19_[CH_3_CH_2_C-(CH_2_O)_3_]}∙13H_2_O
Formula weight	1193.87	1207.89
Crystal system	Monoclinic	Monoclinic
Space group	*C*2/*m*	*C*2/*m*
a (Å)	22.294 (5)	22.170 (1)
b (Å)	10.180 (2)	10.146 (1)
c (Å)	16.394 (4)	16.777 (1)
α (deg.)	90	90
β (deg.)	114.233 (8)	112.257 (2)
γ (deg.)	90	90
V (Å^3^)	3392.6 (12)	3492.5 (3)
Z	4	4
*D_c_* (g cm^−3^)	2.337	2.297
*F* (000)	2352	2384
Reflections coll./unique	24947/4045	21017/3261
*R*_int_	0.0785	0.0315
GOOF on *F^2^*	1.029	1.111
*^a^ R*_1_ [*I* > 2*σ*(*I*)]	0.0468	0.0410
*^b^ wR*_2_ (all data)	0.1154	0.1218
CCDC no.	2206198	2206199

*^a^ R*_1_ = Σ||F_o_| − |F_c_||/Σ|F_o_|. *^b^ wR*_2_ = Σ[*w*(F_o_^2^ − F_c_^2^)^2^]/Σ[*w*(F_o_^2^)^2^]^1/2^.

## Data Availability

Crystallographic data is obtainable free of charge from the Cambridge Crystallographic Data Centre via www.ccdc.cam.ac.uk/data_request/cif (accessed on 9 September 2022).

## Supplementary Materials

The following supporting information can be downloaded at: https://www.mdpi.com/article/10.3390/molecules27217447/s1. Figure S1: Ball-and-stick representation of the cluster comprising of five {VO_6_} and one {MoO_6_} polyhedra, showing a Lindqvist-type structure, and polyhedron representation of inorganic architecture of compound **1** comprising of a mono-lacunary {V_6_} cluster and a {Mo_2_} dimer; Figure S2: Ball-and-stick representation of polyanion of compound **1**, and two triol ligands covalently modified Lindqvist {V_6_} cluster in *cis* and *trans* conformations; Figure S3: The packing model of polyanion of compound **1** along *a* axis, showing a double-layer structure; Figure S4: FT-IR spectra of compounds **1** and **2**; Figures S5 and S6: XPS spectra for Mo and V of compounds **1** and **2**; Figure S7: PXRD patterns of the as-synthesized and simulated for (a) compound **1**, and (b) compound **2**; Figure S8: TGA curves of compounds **1** and **2**; Figure S9: ^1^H NMR spectra of triol ligand TME and compound **1** after its dissolving in water for different amounts of time; Figure S10. ^1^H NMR spectra of triol ligand TMP and compound **2** after its dissolving in water for different amounts of time; Figure S11: Linear fitness of ln(σT) versus 1/T of compounds **1** and **2**; Table S1: Important bond lengths in the cluster of compound **1**; Table S2: Important bond angles in the cluster of compound **1**; Table S3: Important bond lengths in the cluster of compound **2**; Table S4: Important bond angles in the cluster of compound **2**; Table S5: The proton conductivity performance of different POMs.

## Sample Availability

Samples of the compounds **1** and **2** are available from the authors.
