# Supplementary material for: Organic/Inorganic Species Synergistically Supported Unprecedented Vanadomolybdates
Source: Molecules. 2022 Nov 2;27(21):7447. doi: 10.3390/molecules27217447 (PMC9653653; doi:10.3390/molecules27217447)
Supplement: Supplementary file 1 [file molecules-27-07447-s001.zip › molecules-1998169-supplementary.pdf]

Supporting Information

Organic/Inorganic Species Synergistically Supported Unprecedented Vanadomolybdates

Tian Chang, Di Qu, Bao Li,* Lixin Wu

State Key Laboratory of Supramolecular Structure and Materials, College of Chemistry, Jilin University, Changchun 130012, P. R. China

E−mail: libao@jlu.edu.cn

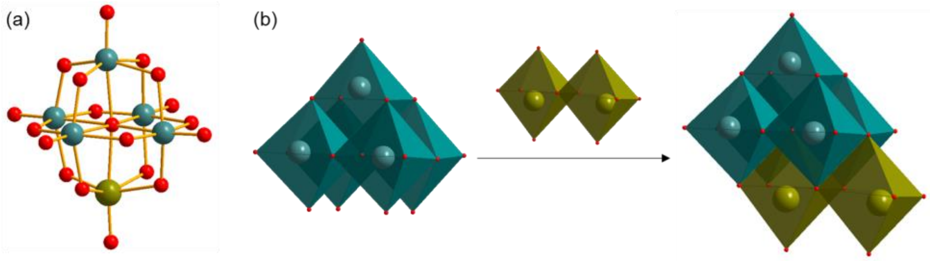

Figure S1. (a) Ball-and-stick representation of the cluster comprising of 5 {VO_6} and 1 {MoO_6} polyhedra, showing a Lindqvist-type structure, (b) Polyhedron representation of inorganic architecture of compound **1** comprising of a mono-lacunary {V_6} cluster and a {Mo_2} dimer. Dark cyan ball: V, olive ball: Mo, red ball: O, dark cyan polyhedron: {VO_6}, olive polyhedron: {MoO_6}.

Table S1. Important bond lengths in the cluster of compound **1**.

	Bond length (Å)		Bond length (Å)
Mo(1)−O(9)	1.683(6)	V(1)−O(1)	1.609(5)
Mo(1)−O(8)#1	1.839(4)	V(1)−O(2)	1.821(3)
Mo(1)−O(8)	1.839(4)	V(1)−O(4)	2.222(5)
Mo(1)−O(10)#1	2.043(3)	V(1)−O(3)	2.016(3)
Mo(1)−O(10)	2.043(3)	V(1)−O(2)#1	1.821(3)
Mo(1)−O(4)	2.304(4)	V(1)−O(3)#1	2.016(3)
Mo(2)−O(13)#1	1.730(4)	V(2)−O(2)	1.851(4)
Mo(2)−O(13)	1.730(4)	V(2)−O(4)	2.276(3)
Mo(2)−O(14)	1.744(5)	V(2)−O(5)	1.848(3)
Mo(2)−O(10)	2.217(3)	V(2)−O(6)	1.599(4)
Mo(2)−O(10)#1	2.217(3)	V(2)−O(7)	1.970(4)
Mo(2)−O(12)	2.303(4)	V(2)−O(8)	1.952(4)
V(3)−O(11)	1.601(3)	V(3)−O(3)	1.956(3)
V(3)−O(12)	2.141(3)	V(3)−O(4)	2.218(3)
V(3)−O(7)	1.736(3)	V(3)−O(10)	1.910(3)

Symmetry transformation used to generate equivalent atoms: #1 x, −y + 2, z

Table S2. Important bond angles in the cluster of compound **1**.

	Bond angle (°)		Bond angle (°)
O(9)−Mo(1)−O(8)	104.80(17)	O(13)#1−Mo(2)−O(13)	105.2(3)
O(8)#1−Mo(1)−O(8)	96.0(2)	O(13)−Mo(2)−O(14)	104.52(17)
O(9)−Mo(1)−O(10)#1	99.70(18)	O(14)−Mo(2)−O(10)	91.76(17)
O(8)#1−Mo(1)−O(10)#1	88.76(15)	O(13)−Mo(2)−O(10)#1	153.83(15)
O(8)−Mo(1)−O(10)#1	152.88(15)	O(10)−Mo(2)−O(10)#1	68.84(17)
O(10)#1−Mo(1)−O(10)	75.70(18)	O(13)#1−Mo(2)−O(12)	89.35(14)
O(9)−Mo(1)−O(4)	173.8(2)	O(14)−Mo(2)−O(12)	156.7(2)
O(8)−Mo(1)−O(4)	79.19(13)	O(10)−Mo(2)−O(12)	69.24(12)
O(10)−Mo(1)−O(4)	75.50(12)	O(13)−Mo(2)−O(10)	89.97(15)
O(1)−V(1)−O(2)	102.64(17)	O(6)−V(2)−O(5)	102.2(2)
O(2)#1−V(1)−O(2)	94.4(2)	O(6)−V(2)−O(2)	103.0(2)
O(1)−V(1)−O(3)	98.71(17)	O(5)−V(2)−O(2)	91.9(2)
O(2)#1-V(1)-O(3)	157.33(15)	O(6)−V(2)−O(8)	100.3(2)
O(2)−V(1)−O(3)	88.21(14)	O(5)−V(2)−O(8)	88.5(2)
O(3)−V(1)−O(3)#1	81.05(17)	O(2)−V(2)−O(8)	156.10(15)
O(1)−V(1)−O(4)	174.6(2)	O(6)−V(2)−O(7)	100.9(2)
O(2)−V(1)−O(4)	80.96(14)	O(5)−V(2)−O(7)	156.49(17)
O(3)−V(1)−O(4)	77.23(12)	O(8)−V(2)−O(7)	82.97(16)
O(11)−V(3)−O(7)	104.23(18)	O(11)−V(3)−O(10)	101.79(17)
O(7)−V(3)−O(10)	97.79(15)	O(11)−V(3)−O(3)	97.20(16)
O(7)−V(3)−O(3)	94.12(15)	O(10)−V(3)−O(3)	154.30(13)
O(11)−V(3)−O(12)	95.26(15)	O(7)−V(3)−O(12)	160.50(15)
O(10)−V(3)−O(12)	78.48(15)	O(3)−V(3)−O(12)	82.70(14)

Symmetry transformation used to generate equivalent atoms: #1 x, −y + 2, z

Table S3. Important bond lengths in the cluster of compound **2**.

	Bond length (Å)		Bond length (Å)
Mo(1)−O(9)	1.679(5)	V(1)−O(1)	1.608(5)
Mo(1)−O(8)	1.840(4)	V(1)−O(2)	1.825(3)
Mo(1)−O(8)#1	1.841(4)	V(1)−O(3)	2.015(3)
Mo(1)−O(10)	2.037(3)	V(1)−O(4)	2.226(4)
Mo(1)−O(10)#1	2.037(3)	V(1)−O(2)#1	1.825(3)
Mo(1)−O(4)	2.302(4)	V(1)−O(3)#1	2.015(3)
Mo(2)−O(13)	1.734(4)	V(2)−O(2)	1.847(3)
Mo(2)−O(13)#1	1.734(4)	V(2)−O(4)	2.269(3)
Mo(2)−O(14)	1.739(5)	V(2)−O(5)	1.844(3)
Mo(2)−O(10)	2.222(3)	V(2)−O(6)	1.608(4)
Mo(2)−O(10)#1	2.222(3)	V(2)−O(7)	1.963(4)
Mo(2)−O(12)	2.292(4)	V(2)−O(8)	1.955(4)
V(3)−O(3)	1.955(3)	V(3)−O(10)	1.909(3)
V(3)−O(4)	2.220(3)	V(3)−O(11)	1.601(4)
V(3)−O(7)	1.735(3)	V(3)−O(12)	2.130(3)

Symmetry transformation used to generate equivalent atoms: #1 x, −y + 1, z

Table S4. Important bond angles in the cluster of compound **2**.

	Bond angle (°)		Bond angle (°)
O(9)−Mo(1)−O(8)	104.43(16)	O(1)−V(1)−O(2)#1	103.03(16)
O(8)−Mo(1)−O(8)#1	95.8(2)	O(2)#1−V(1)−O(2)	94.2(2)
O(9)−Mo(1)−O(10)	100.17(18)	O(1)−V(1)−O(3)	98.45(16)
O(8)−Mo(1)−O(10)	89.07(16)	O(2)#1−V(1)−O(3)	157.26(14)
O(8)#1−Mo(1)−O(10)	152.80(15)	O(2)−V(1)−O(3)	88.14(14)
O(10)−Mo(1)−O(10)#1	174.5(2)	O(3)−V(1)−O(3)#1	81.35(17)
O(8)−Mo(1)−O(4)	79.17(13)	O(1)−V(1)−O(4)	174.2(2)
O(10)−Mo(1)−O(4)	75.51(12)	O(2)−V(1)−O(4)	80.83(13)
O(10)−Mo(1)−O(10)#1	75.2(2)	O(3)−V(1)−O(4)	77.21(12)
O(13)−Mo(2)−O(13)#1	105.0(3)	O(6)−V(2)−O(5)	101.8(2)
O(13)−Mo(2)−O(14)	105.00(17)	O(6)−V(2)−O(2)	102.3(2)
O(13)−Mo(2)−O(10)	152.99(15)	O(5)−V(2)−O(2)	92.4(2)
O(13)#1−Mo(2)−O(10)	90.18(16)	O(6)−V(2)−O(8)	100.5(2)
O(14)−Mo(2)−O(10)	92.01(18)	O(5)−V(2)−O(8)	88.6(2)
O(10)−Mo(2)−O(10)#1	68.03(18)	O(2)−V(2)−O(8)	156.44(15)
O(13)−Mo(2)−O(12)	88.76(14)	O(6)−V(2)−O(7)	101.0(2)
O(14)−Mo(2)−O(12)	156.9(2)	O(5)−V(2)−O(7)	156.60(17)
O(10)−Mo(2)−O(12)	69.05(12)	O(2)−V(2)−O(7)	87.87(15)
O(11)−V(3)−O(7)	104.38(19)	O(11)−V(3)−O(12)	95.99(16)
O(11)−V(3)−O(10)	102.01(17)	O(7)−V(3)−O(12)	159.63(15)
O(7)−V(3)−O(10)	97.23(16)	O(10)−V(3)−O(12)	78.40(15)
O(11)−V(3)−O(3)	94.48(15)	O(3)−V(3)−O(12)	82.60(14)
O(10)−V(3)−O(3)	154.12(15)	O(11)−V(3)−O(4)	171.67(17)

Symmetry transformation used to generate equivalent atoms: #1 x, −y + 1

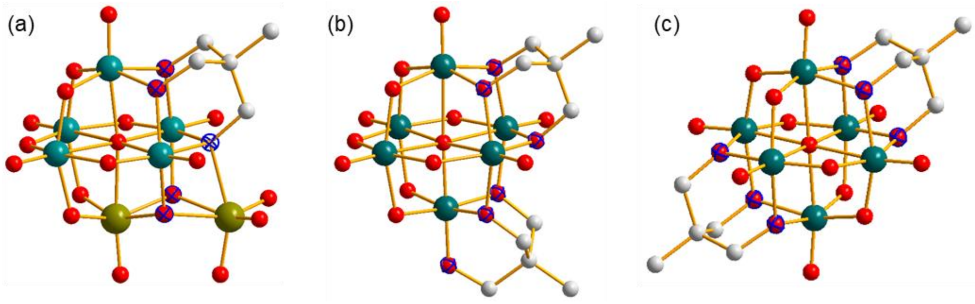

Figure S2. (a) Ball-and-stick representation of polyanion of compound **1**. Red balls with blue circle are shared O atoms by triol ligand or {MoO_6} polyhedron with main cluster. Hollow ball with blue circle is shared O atom by triol ligand and {MoO_6} polyhedron. (b) Two triol ligands covalently modified Lindqvist {V_6} cluster in *cis* conformation. (c) Two triol ligands covalently modified Lindqvist {V_6} cluster in *trans* conformation.

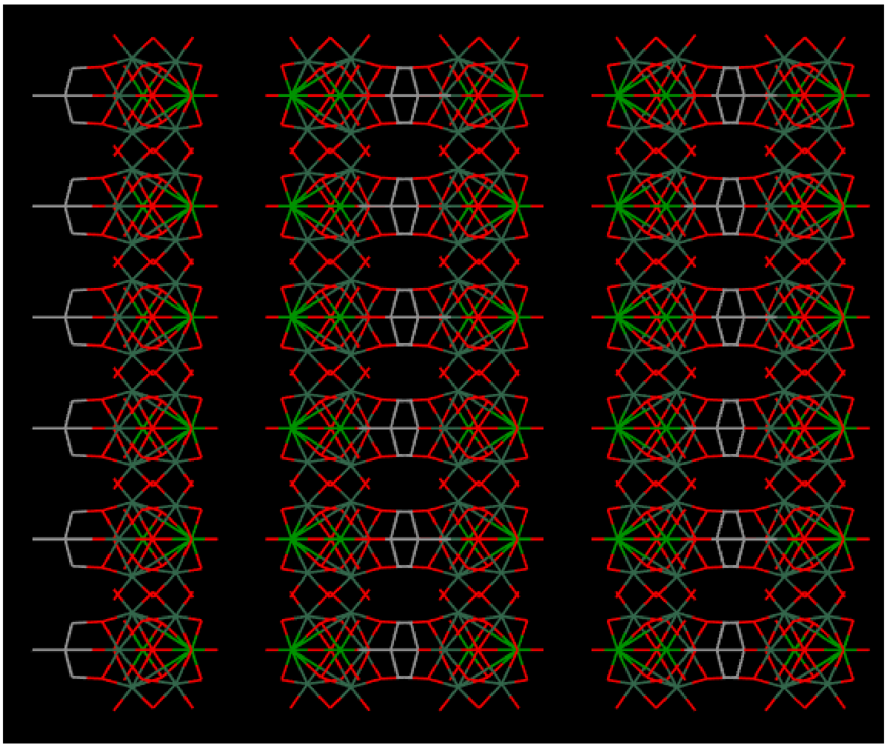

Figure S3. The packing model of polyanion of compound **1** along *a* axis, showing a double layer structure.

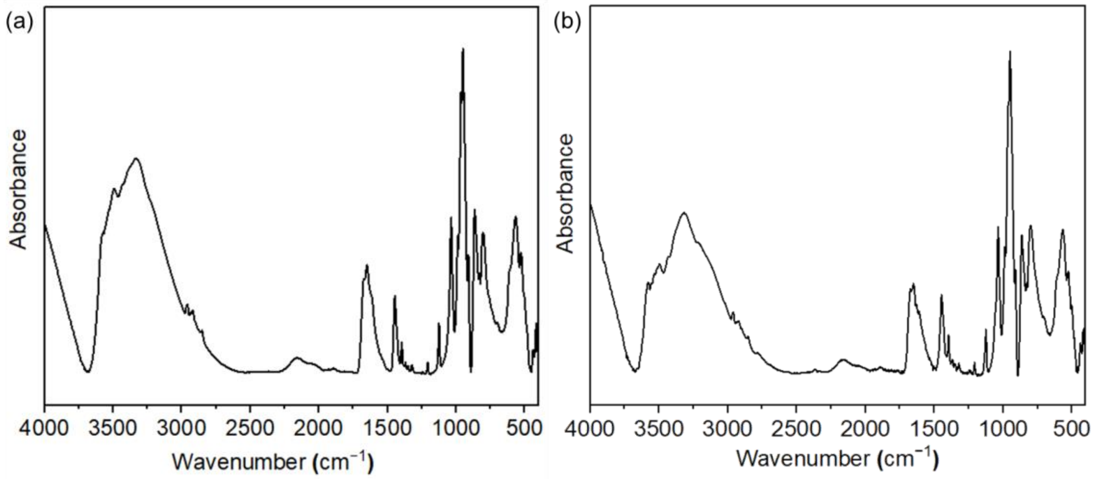

Figure S4. FT-IR spectra of (a) compound **1**, and (b) compound **2**.

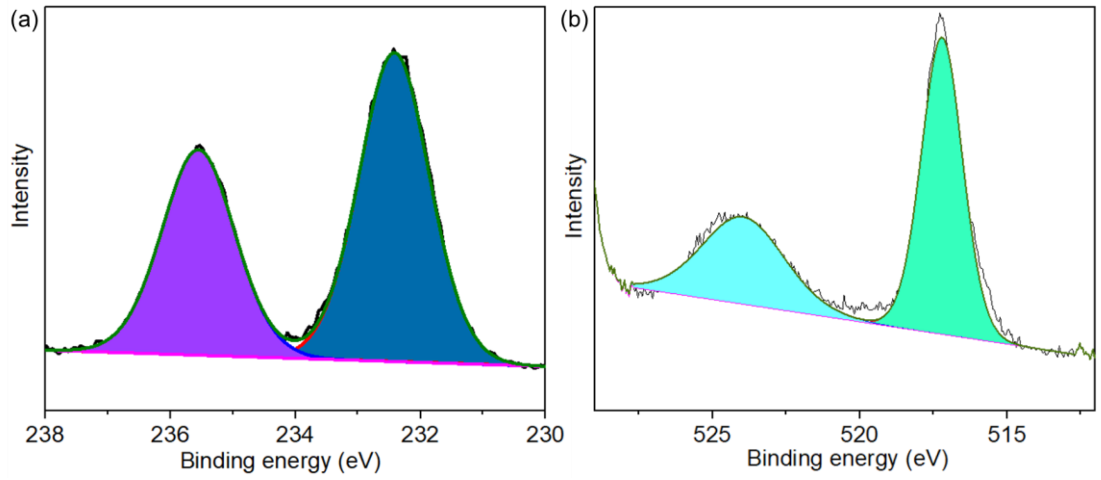

Figure S5. XPS spectra of compound **1** for (a) Mo, and (b) V.

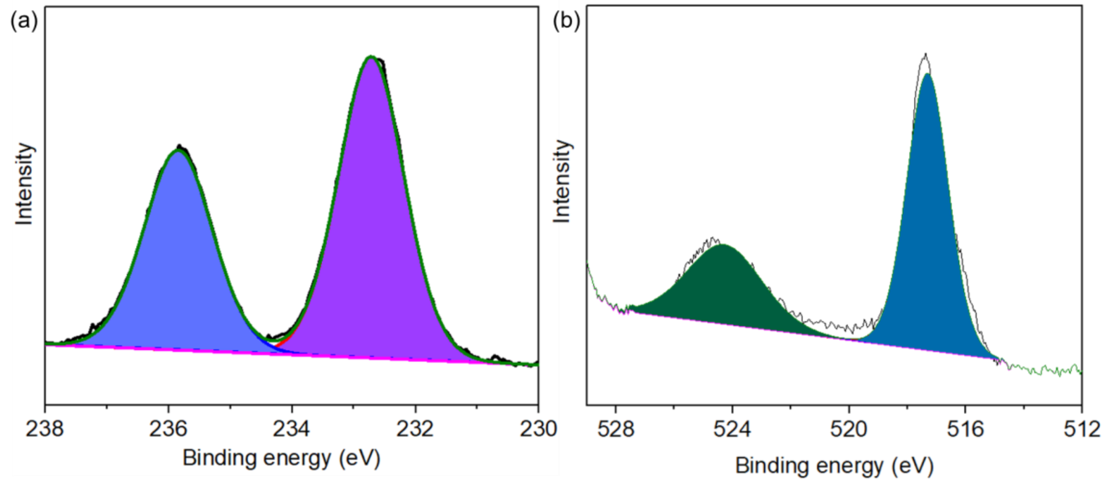

Figure S6. XPS spectra of compound **2** for (a) Mo, and (b) V.

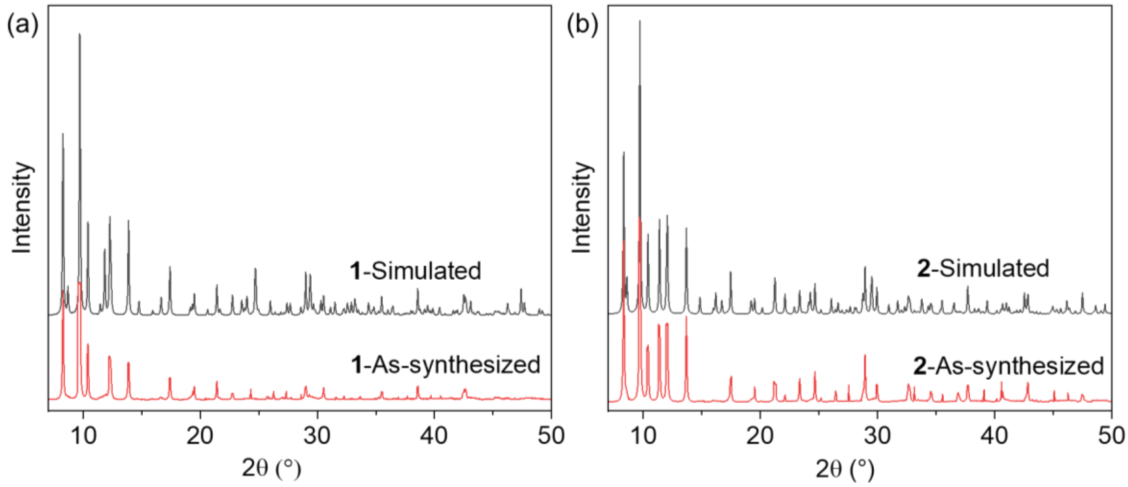

Figure S7. PXRD patterns of the as-synthesized and simulated for (a) compound **1**, and (b) compound **2**.

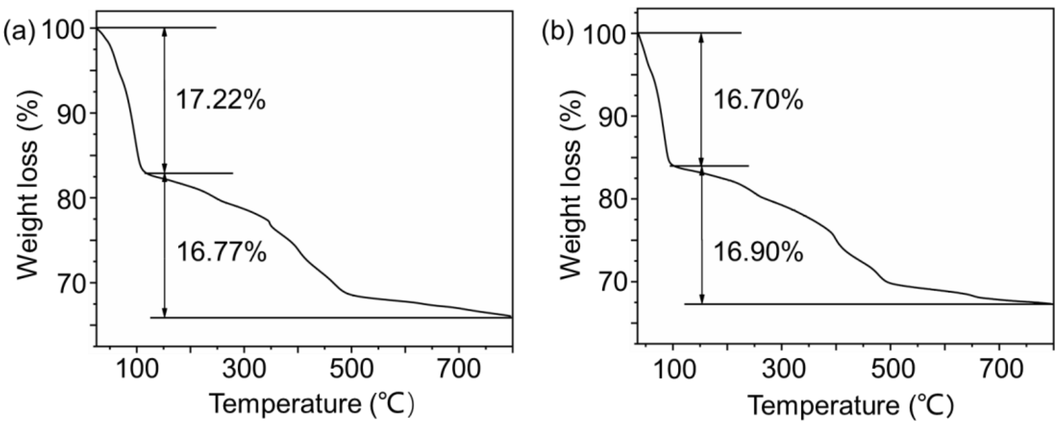

Figure S8. TGA curves of (a) compound **1**, and (b) compound **2**.

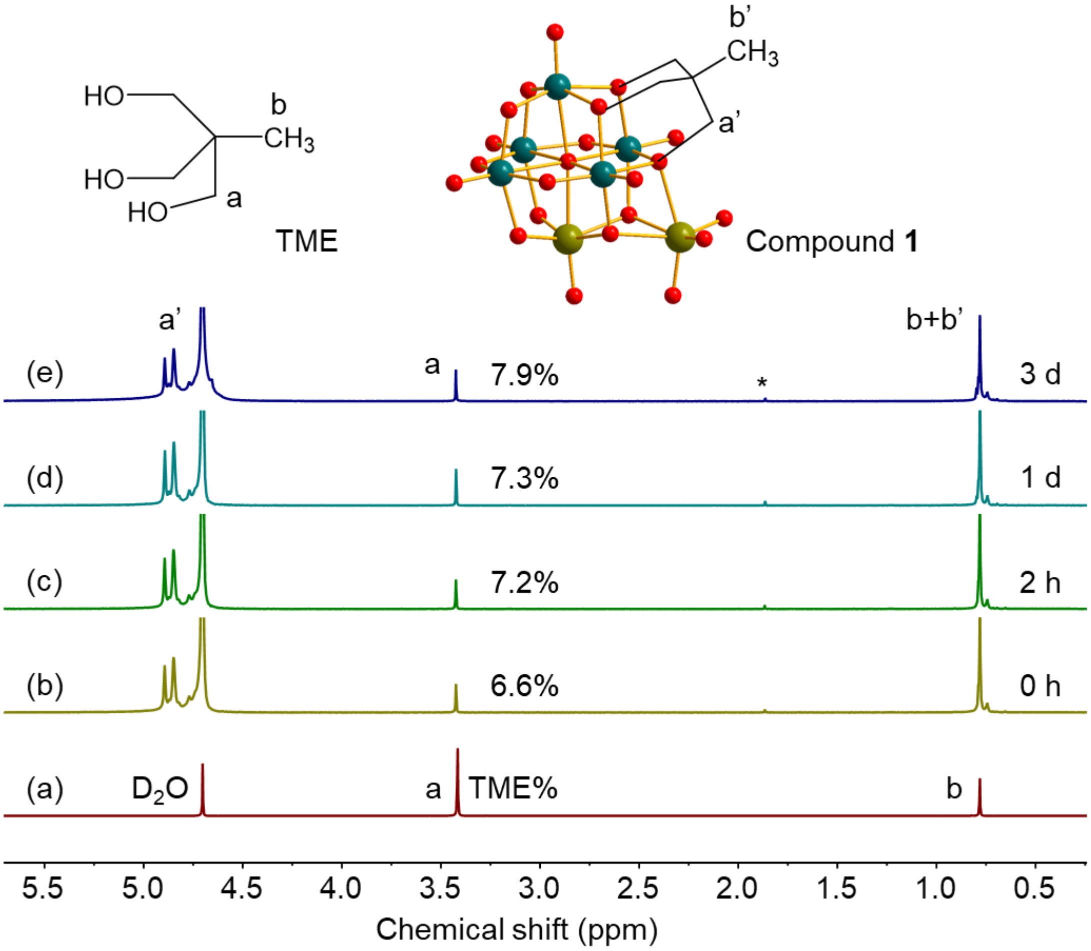

Figure S9. ^{1}H NMR spectra of triol ligand TME and compound **1** after its dissolving in water for different time. * represents unknown species.

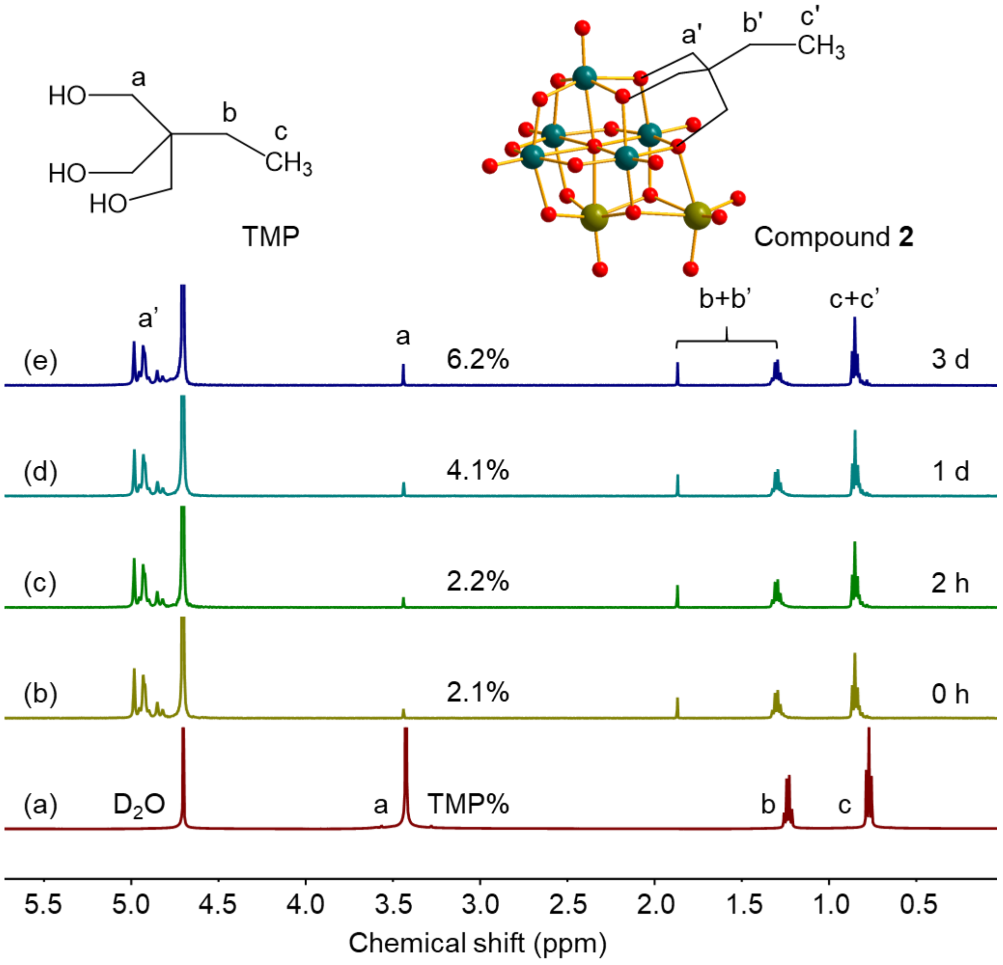

Figure S10. ^{1}H NMR spectra of triol ligand TMP and compound **2** after its dissolving in water for different time.

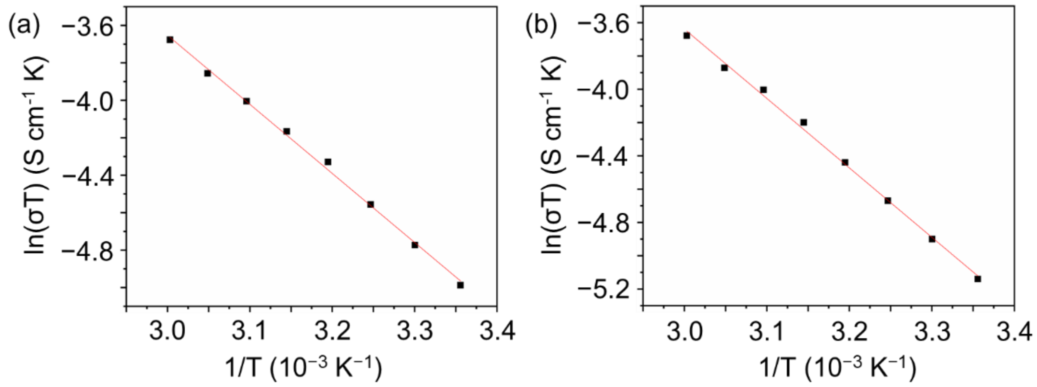

Figure S11. Linear fitness of ln(σT) versus 1/T of (a) compound **1**, and (b) compound **2**.

Table S5. The proton conductivity performance of different POMs.

Compound	T (℃)	Hum.	Proton Conductivity (S cm^{-1})	Ref.
$H_2[Ni(Mo_8O_{26})(bimb)_3]\cdot H_2O$	30	65%	1.71×10^{-7}	[1]
$H_2[Ni(Mo_8O_{26})(bimb)_3]\cdot H_2O$	85	98%	1.03×10^{-2}	
$H_2[Ni(Mo_8O_{26})(bimb)_2]\cdot 4H_2O$	30	65%	6.64×10^{-8}	
$H_2[Ni(Mo_8O_{26})(bimb)_2]\cdot 4H_2O$	85	98%	1.12×10^{-2}	
$[Ni(H_2bibb)_2(H_2O)_3(\alpha\text{-}P_2W_{18}O_{62})]\cdot 7H_2O$	35	98%	1.68×10^{-6}	[2]
$[Co(H_2bibb)_2(H_2O)_3(\alpha\text{-}P_2W_{18}O_{62})]\cdot 10H_2O$	35	98%	1.78×10^{-6}	
$[Cu(H_2bibb)_2(H_2O)_3(\alpha\text{-}P_2W_{18}O_{62})]\cdot 10.5H_2O$	35	98%	1.64×10^{-5}	
$[Cu(H_2bibb)_2(H_2O)](\alpha\text{-}P_2W_{18}O_{62})]\ (H_2bibb)\cdot 0.5H_2O$	35	98%	1.55×10^{-7}	
$K(H_2O)_6[Mn_6(btp)_6(H_2O)_{22}](P_2W_{18}O_{62})_3(Hbtp)_5(btp)_3\cdot 52H_2O$	25	98%	1.07×10^{-5}	[3]
$K(H_2O)_6[Co_6(btp)_6(H_2O)_{22}](P_2W_{18}O_{62})_3(Hbtp)_5(btp)_3\cdot 52H_2O$	25	98%	1.90×10^{-5}	
$[La_{10}Ni_{48}W_{140}Sb_{16}P_{12}O_{568}(OH)_{24}(H_2O)_{20}]^{86-}$	23	100%	2.05×10^{-2}	[4]
$NaH_{15}\{[P_2W_{15}Nb_3O_{62}]_2(4PBA)_2((4PBA)_2O)\}\cdot 53H_2O$	20	98%	1.64×10^{-3}	[5]
$[Cu(en)_2(H_2O)]_2\{[Cu(en)]_4[Cu(en)_2]_5\}\{[Cu(en)_2KNb_{24}O_{72}H_{10}]_2\}\cdot 6en\cdot 70H_2O$	25	98%	3.35×10^{-7}	[6]
$H_3\{[Na_2(H_2O)_2Na_4Fe^{III}_4(H_2O)_4(PO_4)][Na_{0.5}(H_2O)Fe^{II}_{0.5}MoV_4MoV_{I2}(OH)O_{14}(PO_4)_4]_4[Fe^{III}_4(H_2O)_8]\}\cdot 12H_2O$	25	98%	1.07×10^{-3}	[7]
$H_9[Cu(en)(H_2O)_2][Cu(en)_2]_8[Dy(H_2O)_4]_3[Nb_{24}O_{69}(H_2O)_3]_2\cdot 36H_2O$	45	98%	6.67×10^{-5}	[8]
$H_9K[Cu(en)_2(H_2O)]_5[Cu(en)_2]_4[Eu(H_2O)_4]_3[Nb_{24}O_{69}(H_2O)_3]_2\cdot 2en\cdot 45H_2O$	45	98%	2.14×10^{-3}	
$[PMo_{12}O_{40}][H_2PhI]_3[HPhI]\cdot 4H_2O$	40	98%	2.69×10^{-6}	[9]
$LaNH_3CH_2COOCr(OH)_6Mo_6O_{18}$	30	97%	4.55×10^{-5}	[10]
$H_{4.5}NaCo_5(2\text{-}MI)_{15.5}(2\text{-}MI)_3[NaP_5W_{30}O_{110}]\cdot 5H_2O$	70	97%	5.35×10^{-5}	[11]
$H_4[Co(phen)_3]_2[NaO(H_2O)(\tau\text{-}Mo_8O_{26})]_2\cdot 2H_2O$	25	97%	4.82×10^{-4}	[12]
$H_4[TEDA]\ [\gamma\text{-}Mo_8O_{26}]\cdot 3H_2O$	25	97%	3.94×10^{-5}	
$[Cu_4L_2(SiW_{12}O_{40})(OH)(H_2O)_8]\cdot 8H_2O$	30	98%	3.1×10^{-5}	[13]
$La_{0.67}(H_2O)La(H_2O)_6[La(H_2O)_3(SiW_{11}O_{39})]\cdot 7H_2O$	30	97%	6.31×10^{-5}	[14]
$[Ag_8L_5](PMo_{12}O_{40})\cdot(H_2O)_{10}$ (L = 5-Phenyltetrazole)	25	97%	4.79×10^{-6}	[15]
$[Co(bpz)(Hbpz)][Co(SO_4)_{0.5}(H_2O)_2(bpz)]_4[PMo^{VI}_8Mo^{V}_4V^{IV}_4O_{42}]\cdot 13H_2O$	30	98%	2.2×10^{-5}	[16]
$[Ni_2(bpz)(Hbpz)_3(H_2O)_2][PMo^{VI}_8Mo^{V}_4V^{IV}_4O_{44}]\cdot 8H_2O$	30	98%	3.8×10^{-6}	
$\{Na_7[(nBu)_4N]_{17}\}[Zn(P_3Mo_6O_{29})_2]_2\cdot xG$ (G=guest solvent molecules)	25	100%	1.68×10^{-4}	[17]
$Na_4\{V_5Mo_2O_{19}[CH_3C(CH_2O)_3]\}\cdot 13H_2O$	30	98%	1.24×10^{-4}	**This work**
$Na_4\{V_5Mo_2O_{19}[CH_3CH_2C(CH_2O)_3]\}\cdot 13H_2O$	30	98%	1.05×10^{-4}	

Reference:

1. Ren, W.B.; Li, B.; Li, S.; Li, Y.; Gao, Z.; Chen, X.; Zang, H.Y. Synthesis and Proton Conductivity of Two Molybdate Polymers Based on $[Mo_8O_{26}]^{4-}$ Anions. *ChemistrySelect* **2022**, *7*, e202201337, doi:10.1002/slct.202201337.
2. Li, D.; Tan, X.-L.; Chen, L.-L.; Liu, X.-Y.; Li, Y.-M.; Dang, D.-B.; Bai, Y. Four Dawson POM-based inorganic-organic supramolecular compounds for proton conduction, electrochemical and photocatalytic activity. *J. Solid State Chem.* **2022**, *305*, 122694, doi:10.1016/j.jssc.2021.122694.
3. Chen, L.L.; Wu, Y.Y.; Wu, W.W.; Wang, M.M.; Lun, H.J.; Dang, D.B.; Bai, Y.; Li, Y.M. Ultrastable Polyoxometalate-Encapsulated Supramolecular Metal-Organic Nanotubes for Single-Crystal Proton Conduction. *Inorg. Chem.* **2022**, *61*, 8629–8633, doi:10.1021/acs.inorgchem.2c01150.
4. Li, S.R.; Wang, H.Y.; Su, H.F.; Chen, H.J.; Du, M.H.; Long, L.S.; Kong, X.J.; Zheng, L.S. A Giant 3d-4f Polyoxometalate Super-Tetrahedron with High Proton Conductivity. *Small Methods* **2021**, *5*, e2000777, doi:10.1002/smtd.202000777.
5. Li, S.; Zhao, Y.; Knoll, S.; Liu, R.; Li, G.; Peng, Q.; Qiu, P.; He, D.; Streb, C.; Chen, X. High Proton-Conductivity in Covalently Linked Polyoxometalate-Organoboronic Acid-Polymers. *Angew. Chem. Int. Ed.* **2021**, *60*, 16953–16957, doi:10.1002/anie.202104886.
6. Zhu, Z.-K.; Lin, L.-D.; Zhang, J.; Li, X.-X.; Sun, Y.-Q.; Zheng, S.-T. A rare 4-connected neb-type 3D chiral polyoxometalate framework based on $\{KNb_{24}O_{72}\}$ clusters. *Inorg. Chem. Front.* **2020**, *7*, 3919–3924, doi:10.1039/d0qi00927j.
7. Zhang, S.; Lu, Y.; Sun, X.-W.; Li, Z.; Dang, T.-Y.; Zhang, Z.; Tian, H.-R.; Liu, S.-X. Purely inorganic frameworks based on polyoxometalate clusters with abundant phosphate groups: single-crystal to single-crystal structural transformation and remarkable proton conduction. *Chem. Commun.* **2020**, *56*, 391–394, doi:10.1039/c9cc08696j.
8. Zhang, J.; Lai, R.D.; Wu, Y.L.; Zhu, Z.K.; Sun, Y.Q.; Zeng, Q.X.; Li, X.X.; Zheng, S.T. High‐dimensional Polyoxoniobates Constructed from Lanthanide‐incorporated High‐nuclear $\{[Ln(H_2O)_4]_3[Nb_{24}O_{69}(H_2O)_3]_2\}$ Secondary Building Units. *Chem. Asian J.* **2020**, *15*, 1574–1579, doi:10.1002/asia.202000294.
9. Ji, N.-N.; Shi, Z.-Q.; Xie, X.-X.; Li, G. Polyoxometalate-based hydrogen-bonded organic frameworks as a new class of proton conducting materials. *CrystEngComm* **2020**, *22*, 8161–8165, doi:10.1039/d0ce01578d.
10. Yan, T.-T.; Xuan, Z.-X.; Wang, S.; Zhang, X.; Luo, F. Facile one-pot construction of Polyoxometalate-based lanthanide-amino acid coordination polymers for proton conduction. *Inorg. Chem. Commun.* **2019**, *105*, 147–150, doi:10.1016/j.inoche.2019.05.003.
11. Sun, S.; Zhu, L.-J.; Li, K.; Cheng, D.-M.; Li, B.; Wang, Y.-H.; Zang, H.-Y.; Li, Y.-G. A Preyssler-type polyoxometalate-based coordination supramolecule with proton conducting property. *Polyhedron* **2019**, *169*, 84–88, doi:10.1016/j.poly.2019.04.058.
12. Xu, L.; Wang, Z.; Lu, Y.; Yan, T.; Tian, H.; Li, X.; Wang, S.; Sun, X.; Zhang, Z.; Dang, T.; et al. Synthesis and proton conductivity of two novel molybdate polymers. *New J. Chem.* **2018**, *42*, 16516–16522, doi:10.1039/c8nj03781g.
13. Luo, Y.-H.; Yi, L.-Q.; Lu, J.-N.; Dong, L.-Z.; Lan, Y.-Q. A stable polyoxometalate-based porous coordination polymer with high proton conductivity. *CrystEngComm* **2018**, *20*, 6077–6081, doi:10.1039/c8ce00693h.
14. Khan, S.U.; Liu, B.-L.; Akhtar, M.; Du, J.; Peng, J.; Zhao, X.; Xi, W.G.; Zang, H.-Y.; Li, Y.-G. Proton conductive watery channels constructed by in situ generated 3D lanthanide connected

monolacunary polyoxometalate. *Inorg. Chem. Commun.* **2018**, *97*, 187–190, doi:10.1016/j.inoche.2018.09.036.

15. Zhu, M.; Ma, Q.; Zhao, Y.-Z.; Ren, H.-P.; Ding, S.-Y.; Tian, S.-P.; Li, K.-X.; Miao, Z.-C. Facile proton conduction via Keggin anion ··· H_2O H-bonded chain in a Ag^+-5-Phenyltetrazole framework by acid-functionalization. *Inorg. Chem. Commun.* **2017**, *83*, 109–112, doi:10.1016/j.inoche.2017.06.028.
16. Li, J.; Cao, X.L.; Wang, Y.Y.; Zhang, S.R.; Du, D.Y.; Qin, J.S.; Li, S.L.; Su, Z.M.; Lan, Y.Q. The Enhancement on Proton Conductivity of Stable Polyoxometalate-Based Coordination Polymers by the Synergistic Effect of MultiProton Units. *Chem. Eur. J.* **2016**, *22*, 9299–9304, doi:10.1002/chem.201601250.
17. Gao, Q.; Wang, X.L.; Xu, J.; Bu, X.H. The First Demonstration of the Gyroid in a Polyoxometalate-Based Open Framework with High Proton Conductivity. *Chem. Eur. J.* **2016**, *22*, 9082–9086, doi:10.1002/chem.201601233.
